# *Blumea balsamifera* Alleviates Rheumatoid Arthritis by Suppressing Synovial Inflammation via the PI3K/AKT Signaling Pathway: From Chemical Profiling to Molecular Mechanism

**DOI:** 10.3390/ph19030359

**Published:** 2026-02-25

**Authors:** Tianyu Tang, Ke Zhong, Kailang Mu, Yuan Wang, Mengyue Wang, Shan Sha, Xuehua Wu, Zhiwei Wu, Jingli Li, Yanfei Li, Zhanchi Xu, Yuxin Pang

**Affiliations:** 1 College of Pharmacy, Guizhou University of Traditional Chinese Medicine, Guiyang 550025, China; tang13984216529@163.com (T.T.); lionzhongke@163.com (K.Z.); mkl980818@163.com (K.M.); 19108503227@163.com (Y.W.); mywangmoon@163.com (M.W.); sha18932003204@163.com (S.S.); 17694293531@163.com (X.W.); 17685172128@163.com (Z.W.); 18083417719@163.com (J.L.); 15764380153@163.com (Y.L.); xuzhanchi021@gzy.edu.cn (Z.X.); 2 Blumea Research Center, Guizhou University of Traditional Chinese Medicine, Guiyang 550025, China

**Keywords:** *Blumea balsamifera*, ethnopharmacology, rheumatoid arthritis, chemical profiling, PI3K/AKT signaling pathway, synovial inflammation

## Abstract

**Background/Objectives**: *Blumea balsamifera* (L.) DC. (Miao Medicine: Diangd vob bvid), a medicinal plant with a long ethnopharmacological history in Southwest China, is widely used by the Miao, Li, and Zhuang ethnic groups to treat rheumatic diseases. While previous studies indicated that the ethyl acetate fraction of *B. balsamifera* (BBEA) possesses potent anti-inflammatory properties, its specific bioactive material basis and the underlying molecular mechanisms against rheumatoid arthritis (RA) remain elusive. **Methods**: In this study, an integrated strategy combining chemical profiling, network pharmacology, and experimental validation was employed. First, UPLC-Q-Exactive-MS/MS analysis was conducted to characterize the chemical constituents of BBEA. Subsequently, network pharmacology and molecular docking were utilized to predict potential active compounds and core signaling pathways. Finally, the therapeutic effects and mechanisms were validated in vivo using a collagen-induced arthritis (CIA) rat model and in vitro using lipopolysaccharide (LPS)-induced RAW264.7 macrophages. **Results**: A total of 25 active constituents, including Genkwanin and Luteolin, were identified in BBEA via UPLC-Q-Exactive-MS/MS. Network pharmacology analysis predicted that the PI3K/AKT signaling pathway is critical for BBEA’s anti-RA activity, and molecular docking confirmed strong binding affinities between key components (e.g., Genkwanin) and core targets (SRC, AKT1). In vivo experiments demonstrated that BBEA significantly reduced the Arthritis Index (AI) and paw swelling, reversed weight loss, and ameliorated synovial hyperplasia in CIA rats (*p* < 0.05). Furthermore, BBEA markedly downregulated the levels of pro-inflammatory cytokines (*TNF-α*, *IL-1β*, *IL-6*, and *IL-17*) both in serum and synovial tissues. Mechanistically, Western blot analysis verified that BBEA inhibited the phosphorylation of PI3K and AKT in a dose-dependent manner. **Conclusions**: This study systematically reveals that BBEA alleviates RA symptoms and synovial inflammation primarily by inhibiting the PI3K/AKT signaling pathway. These findings provide a scientific basis for the traditional application of *B. balsamifera* and suggest BBEA as a promising candidate for RA therapy.

## 1. Introduction

Rheumatoid arthritis (RA) is a chronic, systemic autoimmune disorder characterized by persistent synovial inflammation, pannus formation, and cartilage destruction, which eventually leads to joint deformity and functional disability [1,2,3,4]. Epidemiological studies indicate that RA affects approximately 1% of the global population, with a disproportionately higher prevalence in females [5,6,7]. Currently, the clinical management of RA relies heavily on non-steroidal anti-inflammatory drugs (NSAIDs), glucocorticoids, and disease-modifying antirheumatic drugs (DMARDs) such as methotrexate (MTX) and biological agents [8,9,10]. While these therapies effectively mitigate symptoms and delay disease progression, their long-term use is often limited by severe adverse effects, including gastrointestinal toxicity, hepatotoxicity, and increased susceptibility to infections [11,12,13,14]. Consequently, there is an urgent need to discover novel therapeutic agents with higher efficacy and lower toxicity from natural resources, particularly from traditional ethnomedicines.

In Southwest China, particularly in Guizhou Province, the unique humid and misty mountainous climate contributes to a high incidence of rheumatic diseases among the indigenous populations. In Miao ethnomedicine, RA is classified as “Mengchongjing” (a wind-type disease), which is believed to stem from the invasion of pathogenic factors such as wind, dampness, and toxins into the meridians and collaterals [15,16]. *Blumea balsamifera* (L.) DC. (Asteraceae), known as “Diangd vob bvid” in the Miao language, has been historically utilized to treat “Mengchongjing” due to its properties of dispelling wind, eliminating dampness, and promoting blood circulation [17]. Interestingly, recent epidemiological surveys have revealed that despite the high environmental risks, the usage of local medicinal herbs like *B. balsamifera* plays a crucial role in managing rheumatic conditions in these ethnic minority regions [18,19]. Our previous preliminary studies have also demonstrated that the crude extracts of *B. balsamifera* exhibit significant anti-inflammatory and antimicrobial activities [20,21].

However, despite its empirical efficacy, the modernization of *B. balsamifera* is hindered by a “black box” problem: its bioactive material basis remains obscure, and the molecular mechanisms underlying its anti-RA effects are poorly understood. Specifically, the ethyl acetate fraction of *B. balsamifera* (BBEA), which is rich in flavonoids and terpenoids [22], shows potential therapeutic value, yet its key active components and target signaling pathways have not been systematically elucidated. This lack of mechanistic insight limits its further clinical translation and quality control.

To bridge this gap, this study adopts an integrated strategy combining chemical profiling, network pharmacology, and experimental validation. First, UPLC-Q-Exactive-MS/MS was employed to comprehensively characterize the chemical constituents of BBEA. Subsequently, network pharmacology and molecular docking were utilized to predict the “multi-component, multi-target” regulatory network. Finally, the therapeutic effects and the predicted mechanism—specifically targeting the PI3K/AKT signaling pathway—were rigorously verified using a collagen-induced arthritis (CIA) rat model and LPS-induced RAW264.7 cells. This study aims to provide a scientific basis for the ethnopharmacological use of *B. balsamifera* and to identify promising candidate fractions for RA treatment.

## 2. Results

### 2.1. Silico Studies of BBEA Against RA

#### 2.1.1. Identification of Potential Therapeutic Targets of BBEA Against RA

**Figure 1:**
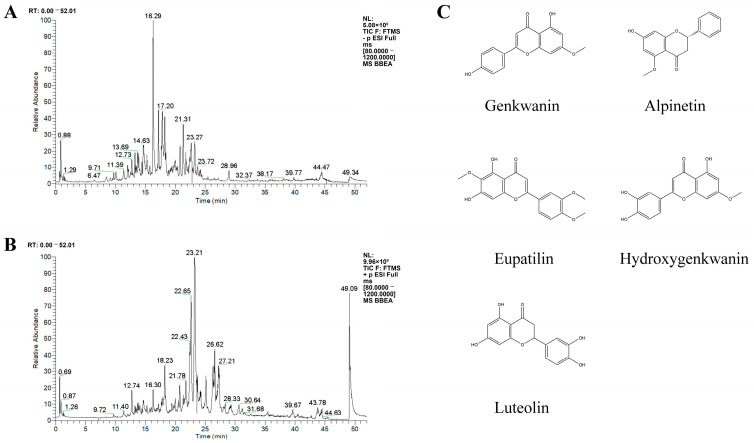
Resulting UPLC-Q-Extractive-MS/MS chromatograms of BBEA. (**A**) Negative ion modes. (**B**) Positive ion modes. (**C**) The core active ingredient chemical structure.

**Figure 2:**
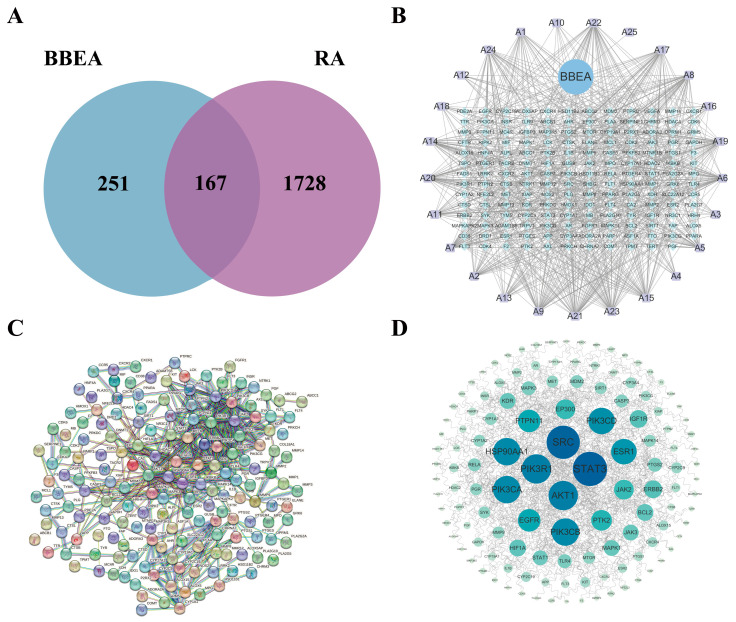
Network pharmacology analysis for screening compounds and pathways of BBEA. (**A**) Venn diagram illustrating the overlap targets of BBEA and RA. (**B**) Compound (BBEA)–target–disease (RA) network. (**C**,**D**) Protein–protein interaction of common genes.

Building on preliminary studies and literature retrieval, 25 chemical components of BBEA were identified (Table 1) (Figure 1). Using the TCMSP and SwissTargetPrediction databases, 251 potential targets of BBEA were acquired following duplicate removal. A total of 1782 RA-related disease targets were recovered based on the GeneCards, DisGeNET, TTD, OMIM, and DrugBank databases. Venn diagram analysis revealed an overlap of 167 common targets between the 251 potential BBEA targets and 1782 RA-related targets, which were defined as the potential therapeutic targets of BBEA against RA (Figure 2A).

#### 2.1.2. Construction of the “Component–Target–Disease” Network

According to the ranking of degree values, the top five essential active elements that were included A21, A23, A22, A17, and A8, while the top five disease targets in order of degree value were *STAT3*, *SRC*, *PIK3R1*, *AKT1*, and *PIK3CA*. These findings indicate that the aforementioned target genes may act as core targets for BBEA against RA (Figure 2B). A total of 167 overlapping target genes were uploaded from the online STRING database to construct a PPI network. The figure displays 167 nodes and 446 edges, with an average degree of nodes of 5.34 and a reliability score of 0.9 (Figure 2C,D).

#### 2.1.3. Enrichment Analysis Reveals the PI3K/AKT Signaling Pathway as a Key Mechanism

**Figure 3:**
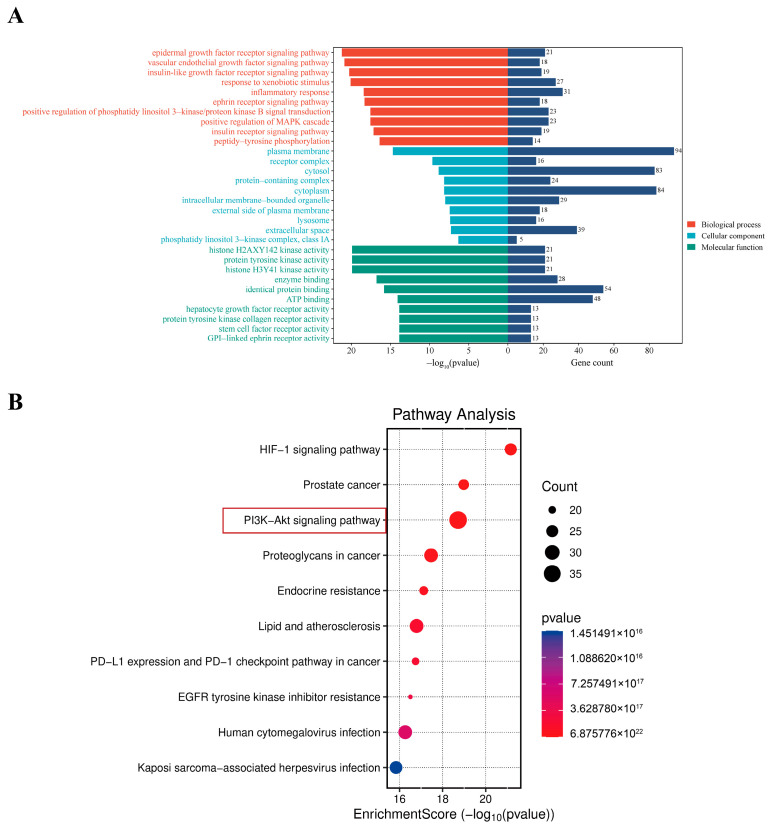
GO and KEGG enrichment analyses. (**A**) GO enrichment analyse. (**B**) KEGG enrichment analyse.

GO enrichment analysis was undertaken on the possible therapeutic targets of BBEA against RA, yielding 675 BPs, 90 CCs, and 203 MFs. The main BP included the epidermal growth factor receptor signaling pathway, the vascular endothelial growth factor signaling pathway and the insulin-like growth factor receptor signaling pathway. Notable CCs included the plasma membrane, the receptor complex, and cytosol. The MF included histone H2AXY142 kinase activity, protein tyrosine kinase activity, and histone H3Y41 kinase activity (Figure 3A). KEGG enrichment analysis showed that the 167 potential targets of BBEA against RA were enriched in 185 signaling pathways (*p* < 0.05, FDR < 0.01). The enrichment bubble plot demonstrated that the core pathways associated with signal transduction and the immune system included the PI3K/AKT pathway, prostate cancer, and the HIF-1 signaling pathway (Figure 3B), suggesting a close association between these pathways and the anti-RA effects of BBEA.

#### 2.1.4. Molecular Docking Validates the Affinity Between Active Components and Core Targets

**Figure 4:**
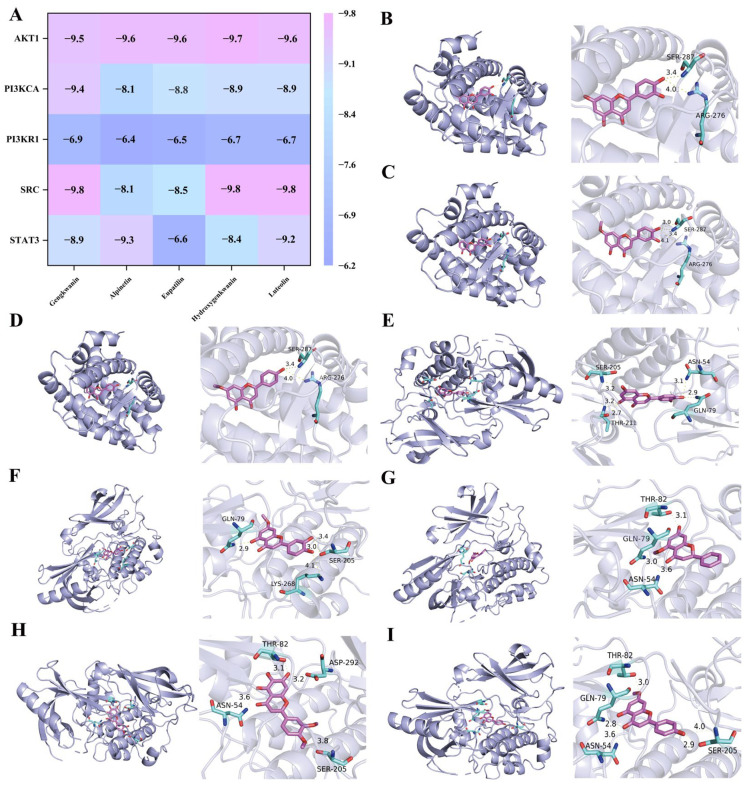
Molecular docking. (**A**) Heat map of molecular docking between major substances and key targets (kcal/mol). The magnitude of binding energy: a larger absolute value corresponds to a redder color, indicating better binding steadiness between the compounds and the proteins. Visualization of molecular docking results. (**B**) *SRC*–Luteolin, (**C**) *SRC*–Hydroxygenkwanin, (**D**) *SRC*–Genkwanin, (**E**) *AKT1*–Luteolin, (**F**) *AKT1*–Hydroxygenkwanin, (**G**) *AKT1*–Alpinetin, (**H**) *AKT1*–Eupatilin, (**I**) *AKT1*–Genkwanin.

The docking results indicated that genkwanin, alpinetin, eupatilin, hydroxygenkwanin, and luteolin exhibited strong binding affinities to the core targets of RA. Among them, genkwanin, hydroxygenkwanin, and luteolin exhibited the highest binding energy with *SRC* (PDB ID: 3 kmr, −9.8 kcal/mol) (Figure 4A). The eight sets of results with the lowest binding energy underwent visual analysis of the target proteins that exhibit strong binding to small molecules affinities using PyMOL software 3.1.4 (Figure 4B–I).

### 2.2. BBEA Attenuates Clinical Symptoms in CIA Rats

**Figure 5:**
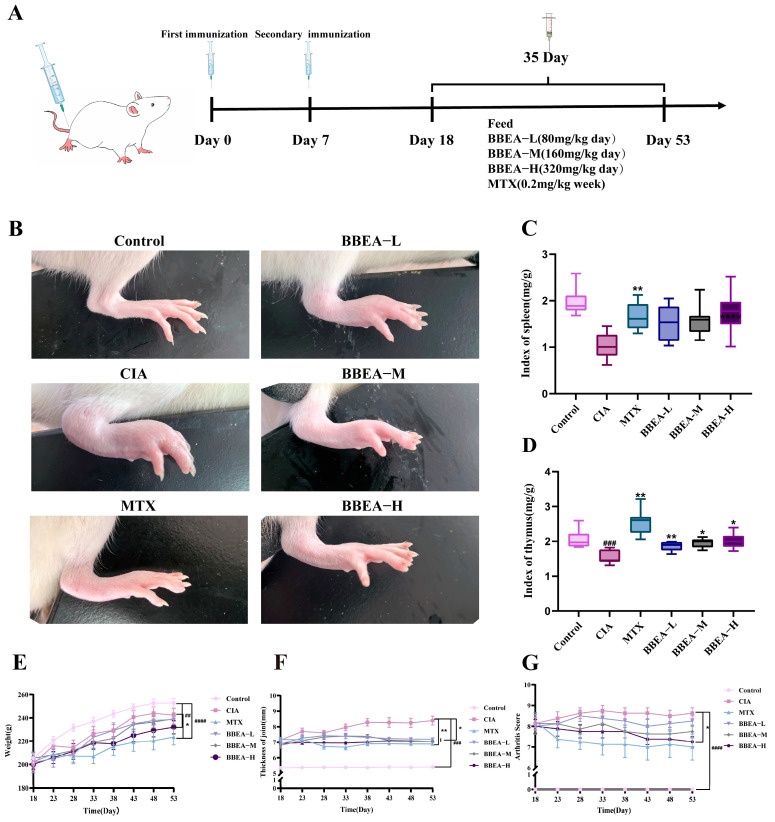
Effect of BBEA on arthritis severity in CIA rats. (**A**) Visual outline of the experimental procedure for CIA rats. (**B**) Representative images of the right hind paws of rats in each group on day 53, (**C**,**D**) Arthritis Index of rats in each group, (**E**) body weight, (**F**) paw thickness, (**G**) AI. Data are displayed as mean ± SD (*n* = 8). Symbols ##, ###, #### indicate compared with the control group; Symbols *, ** indicate *p* < 0.05, *p* < 0.01 compared with the CIA group.

During the entire administration period, the treatments of different groups and the characteristics of the right hind paws of rats are displayed in (Figure 5A,B). The model group displayed swelling of the ankle joints and the entire right hind paw. Following 35 consecutive days of treatment, the symptoms in the treatment groups were alleviated to varying degrees. As shown in Figure 5C, there was a substantial change in the body weight of rats. Relative to the control group, rats in all other groups had lower body weights; the positive group showed a statistically significant variation (*p* < 0.05). During the administration period, the MTX group exhibited a notable therapeutic effect, rapidly reducing the plantar swelling degree and Arthritis Index (AI) of rats (Figure 5D,E). Relative to the control group, the CIA group had a statistically momentous difference in plantar swelling degree during the entire administration period (*p* < 0.001). On day 35, rats were sacrificed, and immune function indicators (thymus index and spleen index) were determined. The thymus index of the CIA group was significantly lower than the control group (*p* < 0.001). As opposed to the CIA group, the spleen index of the MTX group was markedly increased (*p* < 0.01) (Figure 5F,G). With respect to immune organs, the spleens and thymus in the CIA group exhibited atrophy, whereas rats treated with low-dose, medium-dose, and high-dose BBEA (BBEA-L, BBEA-M, BBEA-H) showed upregulated immune function indicators and alleviated splenic swelling. In RA, systemic inflammation can involve extra-articular organs, leading to abnormal immune organ function. Therefore, “immune organ index (organ weight in mg/rat body weight in g)” was used in this study to evaluate the functional status of immune organs. Overall, these findings indicated that BBEA has significant anti-RA properties.

### 2.3. BBEA Ameliorates Histopathological (H&E) Changes in Synovial Tissues

**Figure 6:**
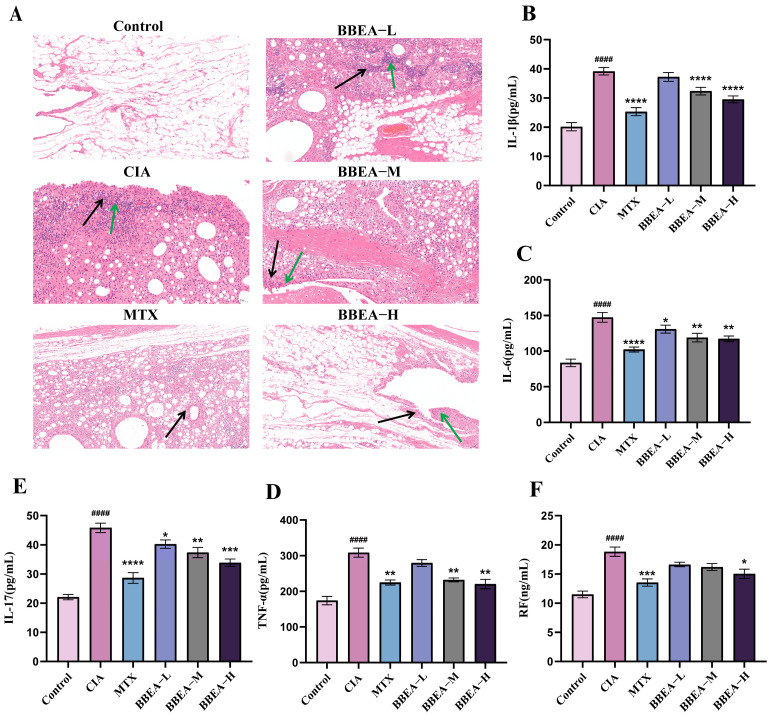
BBEA alleviated pathological features of rheumatoid arthritis in rats. (**A**) Effect of the BBEA on pathological changes in the synovium of the ankle joint in rats (HE, ×200), green arrow represents synovial hyperplasia, black arrow represents inflammatory cell infiltration. The levels of (**B**) *IL-17*, (**C**) *IL-6*, (**D**) *IL-1β*, (**E**) *TNF-α*, (**F**) *RF* in rat serum were determined by ELISA. Data are displayed as mean ± SD (*n* = 8). Symbols #### indicate *p* < 0.000 compared with the control group; Symbols *, **, ***, **** indicate *p* < 0.05, *p* < 0.01, *p* < 0.001, and *p* < 0.0001 compared with the CIA group.

H&E analysis of rat ankle joint specimens demonstrated that the control group maintained well-preserved joint architecture without synovial cell inflammation or swelling (Figure 6A). In contrast with the control group, CIA group exhibited synovial cell swelling, structural disorganization, extensive synovial hyperplasia, massive proliferation of surrounding connective tissues. Additionally, there was an abundance of newly formed blood vessels and substantial infiltration of inflammatory cells mainly including lymphocytes, granulocytes, and macrophages. After treatment with BBEA or MTX, inflammation showed varying degrees of alleviation. In comparison with the CIA group, each BBEA dose group alleviated inflammatory cell infiltration, and most of the joint structures were preserved with considerable integrity.

### 2.4. BBEA Suppresses Systemic Inflammatory Cytokines in Rat Serum

This research quantified the serum levels of pro-inflammatory cytokines *TNF-α*, *IL-1β*, *IL-6*, *IL-17*, and *RF* in rats, revealing an obvious increase in the CIA group compared to others, which were nearly doubly higher than the control group (*p* < 0.0001). After BBEA treatment, the levels of the four inflammatory cytokines and *RF* in the BBEA-H group were markedly decreased (*p* < 0.05). These results imply that BBEA treatment provides systemic therapeutic benefits for RA in the CIA model (Figure 6B–F).

### 2.5. BBEA and Gengkwanin Inhibit Macrophage Activation and NO Release

**Figure 7:**
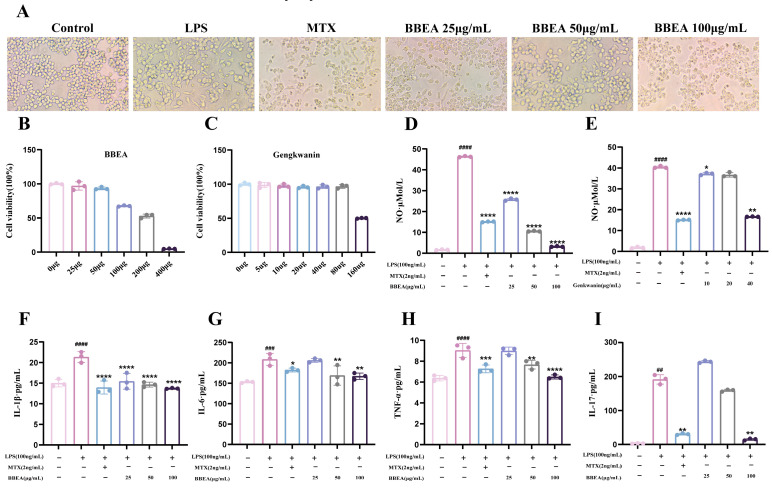
Effects of BBEA and genkwanin on LPS-induced proliferation and NO levels of RAW264.7 cells, and BBEA reduces LPS-induced levels of pro-inflammatory cytokines. (**A**) Cell morphology in different groups. (**B**,**C**) Cell viability. (**D**,**E**) NO levels. (**F**) *IL-1β*, (**G**) *IL-6*, (**H**) *TNF-α*, and (**I**) *IL-17* levels. Data are displayed as mean ± SD (*n* = 3). Symbols ##, ###, #### indicate *p* < 0.05, *p* < 0.01, *p* < 0.001, and *p* < 0.0001 compared with the normal group; Symbols, *, **, ***, **** indicate *p* < 0.05, *p* < 0.01, *p* < 0.001, and *p* < 0.0001 compared with the LPS group.

The effects of different treatments on the morphology of RAW264.7 cells were noted. Cells in the control group were round with smooth edges and lacked pseudopodia (Figure 7A). After LPS stimulation, the morphology of cell in the LPS group changed significantly; most cells transformed from a round to an irregular shape, accompanied by a notable increase in pseudopodia. After treatment with BBEA, an increase in drug concentration led to a shortening and eventual disappearance of cell pseudopodia, while the ratio of round cells gradually elevated. Additionally, the anti-proliferative effects of BBEA and the gengkwanin, screened through network pharmacology based on degree value, on LPS-induced RAW264.7 cells, were investigated. Both BBEA and the genkwanin exhibited strong inhibitory effects on the proliferation of LPS-induced RAW264.7 cells and cell viability was measured by a concentration gradient to explore an appropriate concentration for BBEA and genkwanin treatment (Figure 7B,C). Moreover, the NO content in the supernatant of BBEA and genkwanin were quantified. The results demonstrated that BBEA and genkwanin significantly reduced the NO content in the cell supernatant (BBEA *p* < 0.0001, genkwanin *p* < 0.01)) (Figure 7D,E). BBEA (100 μg/mL) remarkably downregulated the levels of *IL-1β*, *IL-6*, *IL-17*, and *TNF-α* in RAW264.7 cells (Figure 7F–I). In summary, BBEA not only exerts an anti-proliferative effect on LPS-induced RAW264.7 cells but inhibits the release of inflammatory cytokines and NO levels.

### 2.6. BBEA Downregulates mRNA Expression of Pro-Inflammatory Cytokines

**Figure 8:**
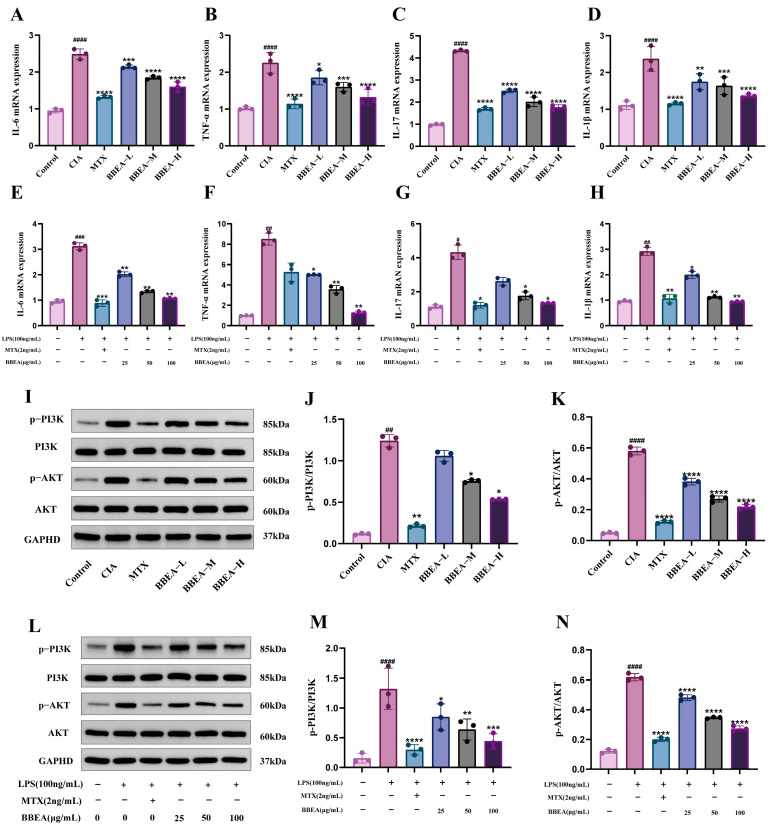
BBEA inhibits mRNA expression levels of inflammatory cytokines and the protein expression contents of PI3K/AKT signaling pathway in RA. mRNA expression of (**A**) *IL-6*, (**B**) *TNF-α*, (**C**) *IL-17*, and (**D**) *IL-1β* genes identified by RT-qPCR in the synovial tissue of rat ankle joints. mRNA expression of (**E**) *IL-6*, (**F**) *TNF-α*, (**G**) *IL-17*, and (**H**) *IL-1β* genes identified by RT-qPCR in LPS-induced RAW264.7 cells. (**I**–**K**) Western blotting was used to detect the protein bands and quantitative densitometric analysis of proteins in the synovial tissue of rat ankle joints. (**L**–**N**) Western blotting was used to detect the protein bands and quantitative densitometric analysis of proteins in LPS-induced RAW264.7 cells. Data are presented as mean ± SD (*n* = 3). Symbols #, ##, ###, ####, *, **, ***, **** indicate *p* < 0.05, *p* < 0.01, *p* < 0.001, and *p* < 0.0001 compared with the normal group and the CIA group, respectively.

We further performed qRT-PCR assays to investigate the effect of BBEA on the expression levels of pro-inflammatory genes in rat joint synovial tissues and LPS-induced RAW264.7 cells. Relative to the LPS group, the BBEA group exhibited a notable decrease in mRNA expression levels of *IL-1β*, *IL-6*, *TNF-α*, and *IL-17* (Figure 8A–H). These findings exhibited that BBEA has a potent anti-inflammatory effect on rat joint synovial tissues and RAW264.7 cells when induced by LPS, which aligns with the results obtained from ELISA.

### 2.7. BBEA Blocks the Activation of the PI3K/AKT Pathway

Western blot analysis revealed that the phosphorylation contents of PI3K and AKT were elevated in synovial tissues from CIA rats and in LPS-treated RAW264.7 cells. However, BBEA treatment significantly suppressed the contents of PI3K and AKT (*p-PI3K*, *p-AKT*) in the cytoplasm of synovial tissues and RAW264.7 cells, without affecting the expression of total PI3K and total AKT (Figure 8I–N). Therefore, these findings indicate that BBEA effectively attenuates the activation of the PI3K/AKT pathway in vivo and vitro.

## 3. Discussion

### 3.1. Material Basis: Flavonoids as Key Effectors

The therapeutic efficacy of any herbal medicine relies on its material basis. In this study, UPLC-Q-Exactive analysis identified 25 compounds in BBEA, predominantly flavonoids. These include luteolin, hydroxygenkwanin, genkwani, eupatilin and alpinetin. Among them, several compounds have previously been confirmed to possess anti-inflammatory and potential anti-arthritic activities, providing an important material basis for the anti-arthritic effects of Artemisia annua extract. Genkwanin, as one of the active ingredients of BBEA, plays a crucial role in preventing the pro-inflammatory mediators and RA by targeting the NF-κB pathway [23]. Alpinetin is effective in protecting Chondrocytes and exhibiting anti-inflammatory effects [24]. Genkwanin, luteolin and hydroxygenkwanin have been confirmed to exert a clear anti-arthritic effect by downregulating the expression of factors such as *NO*, *iNOS*, *TNF-α*, *IL-6*, *IFN-γ* and *IL-2*, and inhibiting the activation of the NF-κB pathway [25]. These components are the flavonoid constituents of this genus of plants, which are easy to separate and identify. They form an important material basis for their pharmacological effects. Our molecular docking results further corroborated this, showing that genkwanin and luteolin spontaneously bind to the active pockets of *SRC* and *AKT1* with high affinity (binding energy <−5.0 kcal/mol). This consistency between in silico predictions and chemical profiling suggests that these flavonoids likely act synergistically to target multiple proteins in the RA network, reflecting the “multi-component, multi-target” characteristic of Traditional Chinese Medicine (TCM).

### 3.2. Mechanism: Targeting the PI3K/AKT Axis to Halt Synovial Hyperplasia

The hallmark of RA pathology is the transformation of the synovium into a hyperplastic, invasive tissue (pannus), often described as a “tumor-like” growth [26]. Moreover, the potential molecular mechanism by which this plant extract exerts its anti-arthritic effect is closely associated with the regulation of several key biomarkers and core target proteins in rheumatoid arthritis. In the pathological progression of RA, these pivotal molecules act as the primary mediators. Firstly, overlapping targets were identified by intersecting 251 predicted BBEA targets with 1728 RA-related genes. Secondly, a PPI network was constructed, which identified top five hub genes (*STAT3*, *SRC*, *PIK3R1*, *AKT1*, and *PIK3CA*). These genes are closely associated with the regulation of signal transduction, protein phosphorylation, cell proliferation, migration, and apoptosis [27]. In this study, PI3K is an important phosphatidylinositol kinase involved in cell growth and skeleton remodeling, and also an important anti-apoptosis regulator. PI3K activation can result in phosphorylation of lipid substrate and activation of downstream AKT [28]. *B. balsamifera* significantly modulated the expression and activation of these core biomarkers, thereby interrupting the pathological cascade and alleviating arthritic symptoms. These findings indicate that the therapeutic effects of *B. balsamifera* are mediated, at least in part, by regulating these disease-related key biomarkers, which further highlights the potential clinical value of this plant extract in the management of rheumatoid arthritis.

GO functional enrichment analysis and KEGG enrichment analysis delineated the potential biological mechanisms through which *B. balsamifera* intervened in RA, demonstrating close associations with key functional modules and the PI3K/AKT signaling pathway. And the results showed that the PI3K/AKT pathway had the highest count value among all potential pathways (Figure 3). The PI3K/AKT signaling pathway is widely confirmed by relevant studies to be a core regulatory pathway in the pathogenesis of RA [29,30,31,32]. Studies have demonstrated that *B. balsamifera* can inhibit the activation of the PI3K/AKT signaling pathway to improve cardiac function of acute myocardial infarction (AMI) model rats [33]. Consistent with these findings, our experimental data provided robust verification: BBEA treatment significantly reduced the phosphorylation levels of PI3K and AKT in both CIA rat synovial tissues and LPS-stimulated macrophages. We postulate that by blocking the PI3K/AKT axis, BBEA disrupts the survival signals of inflammatory cells and inhibits the excessive proliferation of the synovium. This mechanism explains the macroscopic improvements observed in the CIA model, including reduced paw swelling, decreased Arthritis Index, and, most importantly, the amelioration of synovial hyperplasia and pannus formation seen in histopathological sections. These studies indicate the therapeutic value of targeting the PI3K/AKT pathway. Network pharmacology analysis in the present study found that *B. balsamifera*’s regulatory effects were mediated through PI3K/AKT signaling.

### 3.3. New Therapeutic Strategies for Chronic Disease and Cytokine Storm Suppression

Chronic diseases such as rheumatoid arthritis (RA), multiple sclerosis (MS), and type 1 diabetes (T1D) are all mediated by sustained T-cell overactivation, excessive inflammatory cytokine accumulation, and impaired normal regulatory pathways [34]. RA is driven by a cytokine storm, where *TNF-α*, *IL-1β*, and *IL-6* perpetuate inflammation and bone destruction [35,36,37,38]. Our results showed that BBEA by targeting the regulation of the PI3K/AKT axis significantly downregulated the mRNA and protein levels of these key cytokines in both serum and synovial tissues (Figure 8). Through precise targeted therapy, it minimizes harm to the patient’s body while controlling inflammation. In vivo targeted therapy and in vitro targeted therapy are the main categories. For instance, in vivo, isorhamnetin exerts hypoglycemic effects while supporting β-cell integrity through activation of PI3K/AKT and tempering of COX-2-linked lipid-mediator pathways [39]. In vitro, Viviano et al. demonstrated the efficacy of a non-ablative fractional laser for stretch marks, highlighting a trend toward minimally invasive interventions with high safety profiles and improved patient satisfaction [40]. This paradigm aligns with current RA management, where innovative strategies shift from basic inflammation control to targeted, patient-centric approaches that preserve joint integrity and function with minimal systemic disruption. Current clinical treatments like MTX often come with hepatotoxicity. Our study suggests that BBEA, by targeting the PI3K/AKT axis, offers a novel therapeutic strategy that effectively suppresses the cytokine storm (*IL-6*, *TNF-α*) with a potentially better safety profile. This highlights its clinical relevance as a candidate for long-term RA management.

### 3.4. Limitations and Future Directions

Despite these promising findings, this study has limitations. First, while we identified genkwanin as a potential marker compound, its individual pharmacokinetics and contribution to the overall efficacy of BBEA were not separately evaluated in vivo. Second, the results of our experiments indicated that *B. balsamifera* was capable of lowering rheumatoid arthritis symptoms by regulating the PI3K/AKT axis. The present study only focused on the anti-arthritic effect of the current plant extract, and direct comparative data with other well-known anti-rheumatic medicinal plants are still lacking. Common herbal medicines for treating rheumatoid arthritis include *Tripterygium wilfordii* Hook [41], *Periploca forrestii* [42], and *Sinomenium acutum* [43]. In future studies, systematic comparison of efficacy, chemical composition, and mechanism of action between the tested plant and these representative species will be conducted, which will help to further clarify its advantages and application value in the treatment of rheumatoid arthritis. According to the available literature and previous reports, no obvious systemic toxic effects or severe adverse reactions have been documented for this plant extract. However, further systematic and standardized acute and subchronic toxicity studies will still be needed in the future to fully confirm its safety for clinical application. Additionally, we focused on the *PI3K*/*AKT* pathway based on enrichment analysis; however, crosstalk with other pathways (e.g., NF-κB, JAK/STAT) likely exists and warrants further investigation using specific inhibitors or gene knockout models. Future studies will focus on the pharmacokinetic profile of BBEA and validating the direct binding targets using cellular thermal shift assays (CETSA).

Finally, our results from both in vitro cellular experiments and in vivo animal models demonstrated that the extract exerts significant anti-inflammatory and anti-arthritic effects by regulating the PI3K/AKT signaling pathway and its downstream key biomarkers. These results offer reliable experimental evidence for the development of novel therapeutic agents and complementary strategies for rheumatoid arthritis. In addition, the key biomarkers regulated by the extract may represent potential indicators for evaluating disease activity and therapeutic response in clinical practice. To facilitate the clinical translation of this plant extract, future investigations are warranted to identify and characterize the active components, clarify their pharmacodynamic and pharmacokinetic profiles, conduct standardized safety and toxicological assessments, and verify the therapeutic efficacy and safety in well-designed clinical trials. Such step-by-step research will promote the rational development and application of this plant extract as a safe and effective candidate for the clinical treatment of rheumatoid arthritis.

## 4. Materials and Methods

### 4.1. Materials

Bovine type II collagen and nitric oxide (NO) assay kit were obtained from OriLeaf (Shanghai, China). Lipopolysaccharide (LPS), griess reagent and dimethyl sulphoxide (DMSO) were acquired from Solarbio (Beijing, China). Genkwanin was procured from ABPHYTO (Chengdu, China). Commercial enzyme-linked immunosorbent assay (ELISA) kits were obtained from ZCIBIO (Shanghai, China). The cDNA synthesis kit and the primary antibody against *p-PI3K* (AF3242) were obtained from Affinity (Jiangsu, China). Antibodies against AKT (GB15689-100), *p-AKT* (GB150002-100), and PI3K (GB11525-100) were purchased from Servicebio (Wuhan, China).

### 4.2. Preparation of BBEA

*B. balsamifera* was collected from the cultivation base of Guizhou Luodian Aiyuan Ecological Pharmaceutical Development Co., Ltd. (25.49° N, 106.64° E) in July 2024 and authenticated by Prof. Yuxin Pang (School of Pharmacy, Guizhou University of Traditional Chinese Medicine). The sample was preserved in the herbarium of the School of Pharmacy, Guizhou University of Traditional Chinese Medicine, numbered as GZYYXY20250205. Before utilizing, the plant material was placed indoors to naturally dry and ground into fine powder. The *B. balsamifera* powder was soaked in 50% (*v*/*v*) ethanol for 24 h. The filtrate was combined and concentrated at 60 °C. After extractions with equal volumes of ethyl acetate [44], the ethyl acetate fraction of *B. balsamifera* which were then combined and freeze-dried to yield the dried BBEA, with a yield of 5.88%.

### 4.3. Characterization of the Extract by UPLC-Q-Extractive-MS/MS Analysis

The phytochemical composition of BBEA was analyzed by a UPLC-Q-Extractive-MS/MS system consisting of a Dionex Ultimate 3000 RSLC (Thermo Fisher Scientific, Inc. Waltham, MA, USA), a Thermo Scientific Q Exactive Focus mass spectrometer (Thermo Fisher Scientific, Inc.), and a Waters Acquity uplc hss T3 (100 × 2.1 mm, 1.8 μm) (Waters Technologies Shanghai Co., Ltd., Shanghai, China). Gradient elution was performed using water containing 0.1% formic acid (A) and acetonitrile (B) at a flow rate of 0.3 mL/min. The column temperature was maintained at 40 °C, and the injection volume was 3 μL.

### 4.4. Screening of Active Components

The structures of the identified chemical composition were brought into the SwissADME online database (http://www.swissadme.ch/ (accessed on 27 November 2025)), with screening criteria set as gastrointestinal (GI) absorption = High and Druglikeness ≥ 2 “Yes” [45]. TCMSP database (https://www.tcmsp-e.com/load_intro.php (accessed on 27 November 2025)) had OB ≥ 15% and DL ≥ 0.1 [46]. Basic information for each chemical component was searched in the PubChem database (https://pubchem.ncbi.nlm.nih.gov/ (accessed on 27 November 2025)). The SwissTargetPrediction database was utilized to forecast possible protein targets of the components (https://swisstargetprediction.ch/ (accessed on 27 November 2025)). Subsequently, protein names were normalized via the UniProt database (species: *Homo sapiens*), (http://www.uniprot.org/ (accessed on 27 November 2025)) yielding potential therapeutic targets of BBEA.

### 4.5. Acquisition of RA Disease Targets

The GeneCards database (https://www.Genecards.org/ (accessed on 27 November 2025)) was used to retrieve RA-related targets with the criterion of relevance score ≥ 3, as well as DrugBank database (https://www.drugbank.ca/ (accessed on 27 November 2025)), the OMIM database (https://omim.org/ (accessed on 27 November 2025)), and TTD database (https://ttd.idrblab.cn/ (accessed on 27 November 2025)), by searching for “rheumatoid arthritis”. We removed redundant and duplicate targets, and standardized the target names to obtain RA-related targets.

### 4.6. Network Construction

The online plotting tool jvenn (https://jvenn.toulouse.inra.fr/app/example.html (accessed on 27 November 2025)) was used to generate a Venn diagram for BBEA’s therapeutic targets for RA. Additionally, Cytoscape 3.10.0 software was employed to construct a visual network of drug (BBEA)-active components–targets–disease (RA).

### 4.7. Protein–Protein Interaction (PPI) Network Analysis

To build a PPI network, we use a STRING database (https://string-db.org/), set the species as Homo sapiens, and use a confidence threshold of 0.9 or higher. After that, we load this network into Cytoscape version 3.10.0 for additional research. Then, we find out hub genes by evaluating some key topological indexes, especially degree, betweenness and closeness centrality.

### 4.8. GO and KEGG Enrichment Analyses

The potential targets of BBEA for the treatment of RA were submitted to the DAVID database for GO analysis and KEGG signaling pathway analyzing enrichment. Findings from the analysis were uploaded to the Bioinformatics online platform (https://www.bioinformatics.com.cn/) to generate visualizations.

### 4.9. Molecular Docking

Molecular docking simulations were carried out to analyze the binding interactions of key target proteins with active compounds. The lower binding energy between ligand and receptor, and the steadier interaction, with a binding energy of <−5.0 kcal/mol, were defined as the reliable binding [47]. Autodock Vina conducted the molecular docking simulations involving core targets and key compounds from the BBEA, with visualization done using PyMOL 3.4.1 software.

### 4.10. Experimental Animals and Cell Culture

Female Wistar rats aged 6–8 weeks with a weight range of 170–200 g were purchased from Changsha Tianqin Biotechnology Co., Ltd. (Changsha, China) [SCXK (Xiang) 2022-0011]. These rats were housed at Guizhou University of Traditional Chinese Medicine. The animal ethics review was done by the Medical and Laboratory Animal Ethics Committee of Guizhou University of Traditional Chinese Medicine (No: GZY 20250930006). RAW264.7 cells were acquired from Servicebio (Wuhan, China). The cells were kept in DMEM medium supplied with 10% FBS and 1% penicillin-streptomycin (P/S) within a humidified incubator atmosphere of 5% CO_2_ at 37 °C. Cultured cells (1 × 10^4^ cells/well) were planted into 96-well plates and incubated for 24 h. BBEA and genkwanin were prepared in DMSO at concentrations below 0.1% for cell experiments.

### 4.11. Establishment of CIA Model and Drug Administration

Bovine type II collagen (2 mg/mL, mixed in 0.05 mol/L acetic acid) blended with an equivalent volume of complete Freund’s adjuvant (CFA). On day 0, each rat received a hypodermic injection of 0.3 mL of the emulsion in the right hind paw and the tail’s base for primary immunization. Seven days post-primary immunization, booster immunization was administrated via subcutaneous injection of 0.1 mL of the bovine type II collagen emulsion at the tail base [48]. The control group received identical volumes of normal saline at the same anatomical locations and time points as the experimental group. Joint inflammation was assessed on a daily basis throughout the study period. On day 18 after the primary immunization, the Arthritis Index (AI) of collagen-induced arthritis (CIA) rats was evaluated. The AI was scored on a 0–4 scale based on joint manifestations: 0 = no signs of hyperemia or inflammation; 1 = erythema of toe joints without obvious swelling; 2 = slight erythema accompanied by mild swelling of toe joints; 3 = hyperemia and swelling of all tissues below the ankle joint; and 4 = severe swelling involving all joints, comprising the ankle. The total AI score of each rat was the sum of the scores of its right hind paw, and rats with a total AI score > 4 were defined as successfully established CIA models [49]. At the end of each experimental protocol, animals were anesthetized using lsoflurane inhalation.

Forty-eight CIA model rats were selected at random and allocated to six groups with eight rats per group (*n* = 8): model group, MTX group (0.2 mg/kg per week), BBEA low-dose group (BBEA-L: 80 mg/kg per day), BBEA medium-dose group (BBEA-M: 160 mg/kg per day), and BBEA high-dose group (BBEA-H: 320 mg/kg per day). A normal control group (*n* = 8) was also included. The selected *B. balsamifera* doses were determined based on the quantity of crude drugs, and the human and animal doses were converted according to the body surface area normalization method. Rats in each treatment group were intragastrically administered the corresponding dose of BBEA once daily for 35 consecutive days (from day 18 to day 53). Rats in the control and model groups were fed an equivalent amount of 0.9% normal saline. Throughout the entire period, the condition of the ankle joints and the body weight of the rats were monitored.

### 4.12. Assessment of Body Weight, Paw Swelling, and AI

Body weight, paw swelling degree, and AI scores of the rats were recorded every 3 days beginning on day 18. Then, the thickness of the plantar surface on the right hind paw was measured with a vernier caliper, ensuring that the probe gently contacted the plantar skin without applying pressure. Each measurement was conducted in triplicate of each rat, and the average value was recorded. The AI score was assessed by manual evaluation.

### 4.13. Histopathological Examination

Ankle joint tissues were harvested from rats in each group and steeped in a 10% neutral buffered formalin. Following decalcification in 10% EDTA solution, the tissues underwent dehydration, paraffin infiltration, and embedding. Then, the tissue samples were prepared and stained with hematoxylin–eosin (H&E). Pathological alterations in the ankle joint tissues of each group were observed and analyzed under a light microscope.

### 4.14. Thymus and Spleen Indices

On day 35, the rats were anesthetized undergoing sacrifice. The spleen and thymus of each rat were harvested, with adherent fat and connective tissues meticulously removed. Each organ was weighed individually and recorded, followed by the computation of immune organ indices. The index of immunological organs of rats was figured out as

(1) $$\mathrm{Index}\;\mathrm{of}\;\mathrm{Immunological}\;\mathrm{Organs}\;(\%)=\frac{\mathrm{Rat}\;\mathrm{organ}\;\mathrm{weight}\;(\mathrm{mg})}{\text{Rat}\;\text{body}\;\text{weight}\;(g)}$$

### 4.15. Cell Viability Assay

The Cell Counting Kit-8 (CCK-8) assay was utilized to assess the viability of RAW264.7 cells treated with BBEA and genkwanin. Cells were plated in 96-well plates at a density of 2 × 10^4^ cells per well. After 24 h of incubation, cells were treated with the aforementioned compounds at predetermined safe concentrations. After 24 h of co-incubation with the test reagents, CCK-8 solution was added into each well, and an additional 1 h of incubation was performed in the dark. Absorbance at 450 nm was measured using a microplate reader. Cell viability percentage was calculated as

(2) $$\mathrm{Cell}\;\mathrm{viability}\;(\%)=\frac{{\mathrm{OD}}_{\mathrm{treated}}-{\mathrm{OD}}_{\mathrm{blank}}}{{\mathrm{OD}}_{\mathrm{control}}-{\mathrm{OD}}_{\mathrm{blank}}}\times 100\%$$

### 4.16. Nitric Oxide (NO) Assay

The Griess assay was used to quantify nitric oxide (NO) levels in RAW264.7 cells. Cells were cultured and treated as described in Section 4.10. Following collecting 50 μL of cell supernatant into a 96-well plate, 50 μL of Griess Reagent I (S0021S-2) and 50 μL of Griess Reagent II (S0021S-3) were added to each well, with thorough mixing. The absorbance (optical density) at 540 nm was measured at room temperature, and the NO concentration in the samples was calculated based on the standard curve.

### 4.17. Enzyme-Linked Immunosorbent Assay (ELISA)

RAW264.7 cells were added to 6-well plates with a concentration of 6 × 10^5^ cells every well, following a 24 h pretreatment with 100 ng/mL LPS. Then, the cells were treated to BBEA at doses of 25, 50, and 100 μg/mL for another 24 h period. After gathering the culture media, it was centrifuged for ten minutes at 3000 r/min. On the basis of the manufacturer’s instructions of the respective ELISA kits, *TNF-α* (ZC-39024), *IL-1β* (ZC-37974), *IL-6* (ZC-37988), and *IL-17* (ZC-337971) levels were assessed in the cell supernatant. Simultaneously, the levels of these inflammatory mediators and *RF* (ZC-36930) in rat serum were assessed. The absorbance (optical density) at 450 nm was recorded for each well, and the standard curves were used to compute the protein concentrations.

### 4.18. Quantitative Real-Time PCR (qRT-PCR)

The Trizol reagent method was used to derive total RNA from the tissues of rat ankle joints and RAW264.7 cells in the same manner. The extracted overall RNA was used for synthesizing first-strand complementary DNA (cDNA), and qRT-PCR was subsequently performed for detection. The sequences for the primers we designed are offered in Table 2 of the Materials. The internal reference gene was *GAPDH*, and the 2^(−ΔΔCt)^ technique was applied to determine the relative gene expression levels.

### 4.19. Western Blot (WB) Assay

RAW264.7 cells and synovial tissues samples were lysed using RIPA buffer improved with protease and phosphatase inhibitors. After collecting the resulting lysates, the total protein concentration was quantified prior to sodium dodecyl sulfate–polyacrylamide gel electrophoresis (SDS-PAGE). All protein samples were separated by electrophoresis and then transferred onto polyvinylidene fluoride (PVDF) membranes. After sequential incubation with primary antibodies and secondary antibodies, the membranes were visualized using enhanced chemiluminescence (ECL). Protein bands were imaged with a gel imaging system and subjected to densitometric quantitative analysis.

## 5. Conclusions

**Figure 9:**
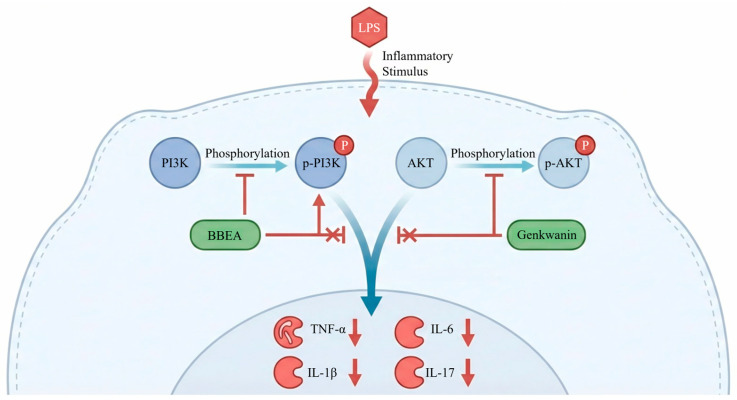
Schematic diagram illustrating the therapeutic mechanism of BBEA against rheumatoid arthritis. BBEA (containing active flavonoids like genkwanin) enters the cell and inhibits the phosphorylation of PI3K and AKT. This blockade suppresses the downstream inflammatory cascade, reduces the secretion of pro-inflammatory cytokines (*TNF-α*, *IL-1β*, *IL-6*, *IL-17*), and inhibits synovial cell proliferation, thereby alleviating joint damage and systemic inflammation in RA.

In summary, this study provides the first systematic evidence deciphering the anti-RA mechanism of the BBEA. We demonstrated that BBEA contains bioactive flavonoids (e.g., genkwanin) that target core RA-related proteins. Therapeutically, BBEA effectively ameliorates joint destruction and systemic inflammation in CIA rats, fundamentally, by inhibiting the phosphorylation activation of the PI3K/AKT signaling pathway and suppressing pro-inflammatory cytokine cascades (Figure 9). These findings not only scientifically validate the traditional use of *B. balsamifera* for rheumatic diseases but also propose BBEA as a promising candidate for the development of novel therapeutic agents against rheumatoid arthritis.

## Figures and Tables

**Table 1:** A total of 25 active components in the ethyl acetate fraction of BBEA.

No.	Name	RT (Time)	Ion Mode	Calc. MW	CAS	OB	DL
A1	Caffeic acid	10.075	[M–H]-	10.075	331–39–5	54.97	0.05
A2	Ferulic acid	12.307	[M–H]-	12.307	537–98–4	39.56	0.06
A3	Xanthoxyline	12.743	[M+H]+	12.743	90–24–4	27.19	0.06
A4	Veratric acid	13.55	[M+H]+	13.55	93–07–2	58.77	0.05
A5	Taxifolin	13.746	[M–H]-	13.746	480–18–2	57.84	0.27
A6	Kaempferol	14.8	[M–H]-	14.8	520–18–3	41.88	0.24
A7	Eriodictyol	17.188	[M–H]-	17.188	552–58–9	71.79	0.24
A8	Luteolin	17.652	[M–H]-	17.652	491–70–3	36.16	0.25
A9	Morin	17.662	[M–H]-	17.662	480–16–0	46.23	0.27
A10	Curcumenol	18.017	[M+H]+	18.017	19431–84–6	91.78	0.13
A11	Ethyl caffeate	18.018	[M–H]-	18.018	102–37–4	103.85	0.07
A12	Curcumol	18.613	[M+H]+	18.613	4871–97–0	109.64	0.13
A13	α-Cyperone	18.953	[M+H]+	18.953	473–08–5	35.53	0.1
A14	Hesperetin	19.865	[M–H]-	19.865	520–33–2	70.31	0.27
A15	Diosmetin	19.925	[M+H]+	19.925	520–34–3	31.14	0.27
A16	Atractylenolide	20.715	[M+H]+	20.715	73069–14–4	47.5	0.15
A17	Hydroxygenkwanin	21.758	[M–H]-	21.758	20243–59–8	36.47	0.27
A18	Isoalantolactone	21.76	[M+H]+	21.76	470–17–7	53.43	0.15
A19	Isorhamnetin	21.774	[M–H]-	21.774	529–40–8	14.13	0.34
A20	Pinocembrin	23.738	[M–H]-	23.738	68745–38–0	46.08	0.18
A21	Genkwanin	23.891	[M–H]-	23.891	437–64–9	37.13	0.24
A22	Eupatilin	25.41	[M+H]+	25.41	22368–21–4	29.39	0.38
A23	Alpinetin	28.548	[M+H]+	28.548	36052–37–6	55.23	0.2
A24	Carnosol	30.606	[M+H]+	30.606	5957–80–2	14.96	0.43
A25	(-)-Caryophyllene oxide	32.237	[M+H]+	32.237	1139–30–6	32.67	0.13

**Table 2:** List of primers used for real-time PCR.

Gene	Species	Forward	Reverse
*IL-1β*	Mouse	GCATCCAGCTTCAAATCTCGC	TGTTCATCTCGGAGCCTGTAGTG
*IL-6*	Mouse	CCCCAATTTCCAATGCTCTCC	CGCACTAGGTTTGCCGAGTA
*TNF-α*	Mouse	CCGTCAGCCGATTTGCTATCT	GCAATGACTCCAAAGTAGACCTG
*IL-17*	Mouse	CTGTGTCTCTGATGCTGTTGCTG	CGTGGAACGGTTGAGGTAGTC
*GAPDH*	Mouse	CCTCGTCCCGTAGACAAAATG	TGAGGTCAATGAAGGGGTCGT
*IL-1β*	Rat	TGTGACTCGTGGGATGATGAC	CCACTTGTTGGCTTATGTTCTGTC
*IL-6*	Rat	GAGTTGTGCAATGGCAATTCTG	ACGGAACTCCAGAAGACCAGAG
*TNF-α*	Rat	CCAGGTTCTCTTCAAGGGACAA	GGTATGAAATGGCAAATCGGCT
*IL-17*	Rat	TCCTCTATTGTCCGCCATGC	ATTTGTATCCCCTCTGCGCC
*GAPDH*	Rat	CTGGAGAAACCTGCCAAGTATG	GGTGGAAGAATGGGAGTTGCT

## Data Availability

The original contributions presented in this study are included in the article. Further inquiries can be directed to the corresponding author.

## Abbreviations

The following abbreviations are used in this manuscript:

RA	Rheumatoid Arthritis
CIA	Collagen-Induced Arthritis
PPI	Protein–Protein Interaction
GO	Gene Ontology
KEGG	Kyoto Encyclopedia of Genes and Genomes
*EGFR*	Epidermal Growth Factor Receptor
*VEGF*	Vascular Endothelial Growth Factor
*IGFR*	Insulin-Like Growth Factor Receptor
ADME	Absorption, Distribution, Metabolism, Excretion
LPS	Lipopolysaccharide
MTX	Methotrexate
DMSO	Dimethyl sulphoxide
DMEM	Dulbecco’s Modified Eagle Medium
*IL-1β*	Interleukin 1
*IL-6*	Interleukin 6
*IL-17*	Interleukin 17
*TNF-α*	Tumor Necrosis Factor α
*RF*	Rheumatoid Factor
ELISA	Enzyme-Linked Immunosorbent Assay
PI3K	Phosphoinositide 3-Kinase
AKT	Protein Kinase B

## References

[1] Smolen J.S., Aletaha D., Barton A., Burmester G.R., Emery P., Firestein G.S., Kavanaugh A., McInnes I.B., Solomon D.H., Strand V. (2018). Rheumatoid arthritis. Nat. Rev. Dis. Primers.

[2] Chen W., Wang J., Xu Z., Huang F., Qian W., Ma J., Wee H.B., Lewis G.S., June R.R., Schafer P.H. (2018). Apremilast Ameliorates Experimental Arthritis via Suppression of Th1 and Th17 Cells and Enhancement of CD4+Foxp3+ Regulatory T Cells Differentiation. Front. Immunol..

[3] Qian X., Zai Z., Tao Y., Lv H., Hao M., Zhang L., Zhang X., Xu Y., Zhang Y., Chen F. (2025). Acidosis regulates immune progression in rheumatoid arthritis by promoting the expression of cytokines and co-stimulatory molecules in synovial fibroblasts. Mol. Med..

[4] Zeng H., Yuan Z., Wu R.-T., Huang Z. (2025). Research progress on complications of rheumatoid arthritis. Front. Immunol..

[5] Yang L., He X., Zhi D., Xue Y., Gong X., Dong K., Tian Y. (2024). Melittin promotes dexamethasone in the treatment of adjuvant rheumatoid arthritis in rats. Front. Pharmacol..

[6] Shen J., Fang Y., Xu N., Chen H., Zhu M., Li D., Chu Z., Sunagawa M., Liu Y., Wang H. (2025). Exploring the mechanism of triptolide inhibiting the motility of fibroblast-like synoviocytes in rheumatoid arthritis via RhoA/Rho-associated kinase axis, based on network pharmacology, molecular docking and molecular dynamics simulations. Front. Pharmacol..

[7] Wei X., Zheng S.G. (2025). Immunomodulatory role of natural products in treating rheumatoid arthritis. Int. Immunopharmacol..

[8] Cheema K.S., Mansour A.B., Raychaudhuri S.P. (2025). What’s new on the horizon for rheumatoid arthritis management. Best Pr. Res. Clin. Rheumatol..

[9] Faison M.N., Davis A.M., Trotter K.C. (2024). Disease-Modifying Drugs for Adult-Onset Rheumatoid Arthritis. JAMA.

[10] Li X., Yang Y., Sun G., Dai W., Jie X., Du Y., Huang R., Zhang J. (2020). Promising targets and drugs in rheumatoid arthritis. Bone Jt. Res..

[11] van Lint J.A., Bakker T., Klooster P.M.T., van Puijenbroek E.P., Vonkeman H.E., Jessurun N.T. (2021). Neuropsychiatric adverse drug reactions associated with low dose methotrexate in rheumatoid arthritis patients. Expert Opin Drug Saf..

[12] Khoroshun K., Bantel C., Hoffmann F., Jobski K. (2025). Methotrexate-related drug reactions on kidneys and liver in rheumatoid arthritis: An analysis of spontaneous reports in EudraVigilance. Arthritis Res. Ther..

[13] Arava S., Uppuluri R.R., Fatima F., Mohiuddin M.Y., Rani A., Kumar D., Challa S., Jonnada S., Purna D.S. (2013). AB0850-HPR Side effect profile in patients with rheumatoid arthritis on leflunomide with and without loading dose. Ann. Rheum. Dis..

[14] Lin Y.-J., Anzaghe M., Schülke S. (2020). Update on the Pathomechanism, Diagnosis, and Treatment Options for Rheumatoid Arthritis. Cells.

[15] Du J., Zhao N., Zhao J., Zhang B. (2006). Classification and Treatment Methods for Rheumatic Diseases in Western Miao Medicine. J. Med. Pharm. Chin..

[16] Li H., Hu X., Luo Z., Lu D., Ma W. (2024). Research Progress of Miao Medicine in the Treatment of Rheumatoid Arthritis. Asia-Pac. Tradit. Med..

[17] Pang Y., Wang D., Fan Z., Chen X., Yu F., Hu X., Wang K., Yuan L. (2014). *Blumea balsamifera*—A Phytochemical and Pharmacological Review. Molecules.

[18] He Y., Hu K., Su J., Chen X., Liu Z., Lu T., Li C., Lu Y. (2020). Epidemiological investigation of resident with rheumatoid arthritis in rural minorities from 20 to 79 years old in Qiannan, Guizhou. Mod. Prev. Med..

[19] Bergstra S.A., Sepriano A., Chopra A., Winchow L.-L., Vega-Morales D., Salomon-Escoto K., Matthijssen X.M.E., Landewé R.B. (2023). Country-level socioeconomic status relates geographical latitude to the onset of RA: A worldwide cross-sectional analysis in the METEOR registry. Ann. Rheum. Dis..

[20] Widhiantara I.G., Jawi I.M. (2021). Phytochemical composition and health properties of Sembung plant (*Blumea balsamifera*): A review. Vet. World.

[21] Wang H., Yuan C., Pang Y. (2019). Antibacterial Activity of Flavonoids from *Blumea balsamifera*. Chin. J. Trop. Crops.

[22] Huang X.-L., Wang D.-W., Liu Y.-Q., Cheng Y.-X. (2022). Diterpenoids from *Blumea balsamifera* and Their Anti-Inflammatory Activities. Molecules.

[23] Gao Y., Liu F., Fang L., Cai R., Zong C., Qi Y. (2014). Genkwanin Inhibits Proinflammatory Mediators Mainly through the Regulation of miR-101/MKP-1/MAPK Pathway in LPS-Activated Macrophages. PLoS ONE.

[24] Gao Y., Wang S., He L., Wang C., Yang L. (2020). Alpinetin Protects Chondrocytes and Exhibits Anti-Inflammatory Effects via the NF-κB/ERK Pathway for Alleviating Osteoarthritis. Inflammation.

[25] Sun Y.-W., Bao Y., Yu H., Chen Q.-J., Lu F., Zhai S., Zhang C.-F., Li F., Wang C.-Z., Yuan C.-S. (2020). Anti-rheumatoid arthritis effects of flavonoids from *Daphne genkwa*. Int. Immunopharmacol..

[26] Nygaard G., Firestein G.S. (2020). Restoring synovial homeostasis in rheumatoid arthritis by targeting fibroblast-like synoviocytes. Nat. Rev. Rheumatol..

[27] Li X., Wang Y. (2020). Cinnamaldehyde Attenuates the Progression of Rheumatoid Arthritis through Down-Regulation of PI3K/AKT Signaling Pathway. Inflammation.

[28] Ruicci K.M., Plantinga P., Pinto N., Khan M.I., Stecho W., Dhaliwal S.S., Yoo J., Fung K., MacNeil D., Mymryk J.S. (2019). Disruption of the RICTOR/mTORC2 complex enhances the response of head and neck squamous cell carcinoma cells to PI3K inhibition. Mol. Oncol..

[29] Malemud C.J. (2015). The PI3K/Akt/PTEN/mTOR Pathway: A Fruitful Target for Inducing Cell Death in Rheumatoid Arthritis?. Future Med. Chem..

[30] Joshi L., Agnihotri P., Saquib M., Chakraborty D., Sarkar A., Choudhary B., Kumar V., Biswas S. (2025). The role of ITIH4 in regulating the PI3K/Akt signalling in rheumatoid arthritis. Rheumatology.

[31] Qi W., Lin C., Fan K., Chen Z., Liu L., Feng X., Zhang H., Shao Y., Fang H., Zhao C. (2019). Hesperidin inhibits synovial cell inflammation and macrophage polarization through suppression of the PI3K/AKT pathway in complete Freund’s adjuvant-induced arthritis in mice. Chem. Interact..

[32] Shao W., Liu F., Zhu L., Qian W., Meng Q., Zhang A., Jin S., Lu J., Yan S.G. (2024). Ferroportin inhibits the proliferation and migration of fibroblast-like synoviocytes in rheumatoid arthritis via regulating ROS/PI3K/AKT signaling pathway. Eur. J. Pharmacol..

[33] Lv Y.J., Wang Y.C., Feng Y.D., Shi Z.F., Han L., Zhang X.Q. (2023). Study on the pharmacodynamics and mechanism of *Blumea balsamifera* total flavonoids against acute myocardial infarction model rats. China Pharm..

[34] Girela M., Gupta D., Grattoni A., Chua C.Y.X. (2025). Targeted immunomodulation for chronic diseases through advanced delivery platforms. Expert Opin. Drug Deliv..

[35] McInnes I.B., Schett G. (2011). The Pathogenesis of Rheumatoid Arthritis. N. Engl. J. Med..

[36] Kieler M., Hofmann M., Schabbauer G. (2021). More than just protein building blocks: How amino acids and related metabolic pathways fuel macrophage polarization. FEBS J..

[37] Kondo N., Kuroda T., Kobayashi D. (2021). Cytokine Networks in the Pathogenesis of Rheumatoid Arthritis. Int. J. Mol. Sci..

[38] Loh C., Park S.-H., Lee A., Yuan R., Ivashkiv L.B., Kalliolias G.D. (2019). TNF-induced inflammatory genes escape repression in fibroblast-like synoviocytes: Transcriptomic and epigenomic analysis. Ann. Rheum. Dis..

[39] Li L., Li J., Ren J., Yao J. (2025). Isorhamnetin Exhibits Hypoglycemic Activity and Targets PI3K/AKT and COX-2 Pathways in Type 1 Diabetes. Nutrients.

[40] Viviano M.T., Provini A., Mazzanti C., Nisticò S.P., Patruno C., Cannarozzo G., Bennardo S., Fusco I., Bennardo L. (2022). Clinical Evaluation on the Performance and Safety of a Non-Ablative Fractional 1340 nm Laser for the Treatment of Stretch Marks in Adolescents and Young Adults: A Case Series. Bioengineering.

[41] Wang Y., Yan J., Zhang Z., Chen M., Wu X., Ma S. (2022). Immunosuppressive Sesquiterpene Pyridine Alkaloids from *Tripterygium wilfordii* Hook. f.. Molecules.

[42] Liu Y., Li M., He Q., Yang X., Ruan F., Sun G. (2016). *Periploca forrestii* Saponin Ameliorates Murine CFA-Induced Arthritis by Suppressing Cytokine Production. Mediat. Inflamm..

[43] Luo J.-F., Yu Y., Yao Y.-D., Zhang C., Liu J.-X., Lio C., Qian H.-B., Zhou H. (2026). Sinomenine treats Rheumatoid Arthritis by regulating IL-6 Gene Promoter Methylation. Phytomedicine.

[44] Zhou L., Xiong Y., Chen J., Zhang J., Hao X., Gu W. (2021). Study on the chemical constituents from the aerial parts of *Blumea balsamifera* DC. and their antioxidant and tyrosinase inhibitory activities. Nat. Prod. Res. Dev..

[45] Chen Y., Huang Y., Su A., Mo J., Feng W., Liao W., Zhu S., Wang L. (2023). Exploration on Action Characteristics of the Stems and Leaves of Melicope ptelefolia on the Treatment of Rheumatoid Arthritis Based on Network Pharmacology and Molecular Docking. Tradit. Chin. Drug Res. Clin. Pharmacol..

[46] Huang J., Tang H., Cao S., He Y., Feng Y., Wang K., Zheng Q. (2017). Molecular Targets and Associated Potential Pathways of Danlu Capsules in Hyperplasia of Mammary Glands Based on Systems Pharmacology. Evid.-Based Complement. Altern. Med..

[47] Xu S., Yu Y., Xie Q., Liu X., Zhang A., Tang H., Zhu Z., Bian X., Guo L. (2025). Revealing the molecular mechanism of Buzhong Yiqi Decoction for tendon bone healing on the basis of network pharmacology, molecular docking and experimental validation. J. Ethnopharmacol..

[48] Yao L., Cheng S., Yang J., Xiang F., Zhou Z., Zhang Q., Pang Y., Zhou W., Abliz Z. (2022). Metabolomics reveals the intervention effect of Zhuang medicine Longzuantongbi granules on a collagen-induced arthritis rat model by using UPLC-MS/MS. J. Ethnopharmacol..

[49] Chi P., Zhang H., Chen Y., Xie J., Ayixianmuguli Y., Wu C., Liu M. (2025). Investigating the underlying mechanisms of the ethanol extract of saussureae involucratae herba in anti-rheumatoid arthritis effect based on sphingolipidomics. Front. Pharmacol..

